# CONCURRENT VISION AND HEARING IMPAIRMENTS, SOCIAL ISOLATION, DEPRESSION, AND ANXIETY: FINDINGS FROM THE NHATS

**DOI:** 10.1093/geroni/igad104.0529

**Published:** 2023-12-21

**Authors:** Shu Xu, Alison Huang, Yunshu Zhou, Joshua Ehrlich

**Affiliations:** University of Massachusetts, Boston, Boston, Massachusetts, United States; Johns Hopkins Bloomberg School of Public Health, Baltimore, Maryland, United States; University of Michigan, Ann Arbor, Michigan, United States; University of Michigan, Ann Arbor, Michigan, United States

## Abstract

Sensory impairments are contributors to poor health for older adults, and the likelihood of having more than one sensory impairment increases with age. There has been little research on population-level associations of visual and hearing function with social and mental health. This study examined the associations between objectively measured hearing and vision impairment, social isolation, depression, and anxiety among older US adults. Using nationally representative data from the 2021 National Health and Aging Trends Study, we conducted cross-sectional analysis among adults aged >70 (n=2,520). Visual impairment (VI) was defined as distance (<20/40), near (<20/40), and/or contrast sensitivity (>1SD below mean) impairment. Hearing impairment (HI) was defined as a better ear pure-tone average >25 decibel hearing level. Respondents with VI and HI were identified as having dual sensory impairment (DSI). Social isolation was assessed by living arrangement, core network size, religious attendance, and social participation. Depressive and anxiety symptoms were measured with the PHQ-2 and GAD-2, respectively. Weighted sample characteristics showed that 25.53% of older adults had DSI. Logistic regression analyses revealed that, compared to those without HI or VI, older adults with DSI were more likely to report social isolation (OR=1.71, 95% CI=1.12-2.60), have depression (OR=4.57, 95% CI=2.34-8.92), and anxiety (OR=2.65, 95% CI=1.36-5.17). Associations with DSI were stronger than for single impairments. All models controlled for age, gender, education, race/ethnicity, marital status, number of chronic conditions. Findings suggested that older adults experiencing DSI are at greater risk of having social isolation and emotional distress.

